# Triolein emulsion enhances temozolomide brain delivery: an experimental study in rats

**DOI:** 10.1080/10717544.2021.1998247

**Published:** 2021-11-08

**Authors:** Won-Bae Seung, Seung Heon Cha, Hak Jin Kim, Seon Hee Choi, Juho Lee, Dongmin Kwak, Hyunwoo Kim, Jin-Wook Yoo, Yong-Woo Kim, Sang Kyoon Kim, Da-Sol Lee

**Affiliations:** a Department of Neurosurgery, Dongguk University College of Medicine, Dongguk University Gyeongju Hospital, Gyeongju, South Korea; b Department of Neurosurgery, SMG Yeonse Hospital, Changwon, South Korea; c College of Medicine, Pusan National University, Busan, Korea; d Biomedical Research Institute, Pusan National University Hospital, Busan, South Korea; e College of Pharmacy, Pusan National University, Busan, South Korea; f Pusan National University Yangsan Hospital, College of Medicine, Pusan National University, Busan, South Korea; g Laboratory Animal Center, Daegu-Gyeongbuk Medical Innovation Foundation, Daegu, Korea

**Keywords:** Triolein emulsion, temozolomide, BBB, drug delivery, liquid chromatography

## Abstract

**Purpose:**

To evaluate the enhancement of temozolomide (TMZ) delivery in the rat brain using a triolein emulsion.

**Materials and Methods:**

Rats were divided into the five groups as following: group 1 (negative control), group 2 (treated with triolein emulsion and TMZ 20 mg/kg), and group 3 (TMZ 20 mg/kg treatment without triolein), group 4 (treated with triolein emulsion and TMZ 10 mg/kg), and group 5 (TMZ 10 mg/kg treatment without triolein). Triolein emulsion was infused into the right common carotid artery. One hour later, the TMZ concentration was evaluated quantitatively and qualitatively using high-performance liquid chromatography (HPLC-MS) and desorption electrospray ionization mass spectrometry (DESI-MS) imaging, respectively. The concentration ratios of the ipsilateral to contralateral hemisphere in each group were determined and the statistical analysis was conducted using an unpaired *t*-test.

**Results:**

Quantitatively, the TMZ concentration ratio of the ipsilateral to the control hemisphere was 2.41 and 1.13 in groups 2 and 3, and were 2.49 and 1.14 in groups 4 and 5, respectively. Thus, the TMZ signal intensities of TMZ in group 2 and 4 were statistically high in the ipsilateral hemispheres. Qualitatively, the signal intensity of TMZ was remarkably high in the ipsilateral hemisphere in group 2 and 4.

**Conclusions:**

The triolein emulsion efficiently opened the blood-brain barrier and could provide a potential new strategy to enhance the therapeutic effect of TMZ. HPLC-MS and DESI-MS imaging were shown to be suitable for analyses of enhancement of brain TMZ concentrations.

## Introduction

The blood–brain barrier (BBB) prevents noxious substances from penetrating the brain parenchyma covering the vessel and is a major obstacle for drug delivery to the central nervous system. The prognosis of patients with primary brain tumors remains poor because the BBB acts as a physiological barrier that limits approximately 98% and 100% of small- and large-molecule drugs, respectively, from reaching the parenchymal tissue (Pardridge, 2007). Although considerable progress has been made in the development of therapeutic techniques, such as surgery, radiotherapy, photodynamic therapy, and chemotherapy, the clinical outcome of patients with gliomas remains poor with a 5-year survival rate of <3% for those with glioblastoma (Catuogno et al., 2012).

The heterogeneous integrity of the BBB poses a challenge for drug delivery across the BBB (Abbott, 2013). Generally, glioma progresses when the permeability of the BBB is enhanced compared to that of normal brain tissue (Cruceru et al., 2013). However, the BBB in peripheral glioma remains intact because tumor cells that have escaped migrate to the surrounding brain parenchyma, resulting in a highly refractory nature of the malignant glioma within a 2–3 cm margin of the surgical resection cavity (Veringa et al., 2013; Zhan & Lu, 2012).

Injecting a triolein emulsion into the carotid artery immediately opens the BBB temporarily and reversibly via tight junctions, resulting in increased vascular permeability (Kim et al., 2004; Ryu et al., 2010; Sol et al., 2017). 8-Carbamoyl-3-methyl-imidazo-[5,1-d]-1,2,3,5-tetrazin-4-(3H) (temozolomide, TMZ; SCH 52365), an alkylating agent and imidazotetrazine derivative that exhibits broad-spectrum antitumor activity against murine tumors, is used in the treatment of malignant gliomas (Ruggiero et al., 2006; Stevens et al., 1984).

TMZ is rapidly degraded to its active metabolite, 5-(3-methyltriazen-1-yl) imidazole-4-carboxamide (MTIC), which then rapidly destroys the inactive derivatives, n-5-aminoimidazole-4-carboxamide and methyldiazonium cation (Denny et al., 1994). For the estimation of TMZ, chromatographic and spectrophotometric methods, such as high-performance liquid chromatography (HPLC) with ultraviolet detection and LC coupled with tandem mass spectrometry (MS/MS) are the most frequently used techniques. TMZ has been extracted from biological samples using liquid–liquid extraction or solid-phase extraction (Estlin et al., 1998; Meany et al., 2009).

Desorption electrospray ionization (DESI)-MS imaging of biological tissues is an efficient and highly sensitive MS ionization technique for imaging lipids and metabolites from biological tissues (Eberlin et al., 2011). DESI is clinically applicable because it enables the analysis of biomolecules in the x and y directions via spraying of charged droplets and provides chemical information that can be displayed as two-dimensional (2 D) images (Agar et al., 2011).

Increasing the vascular permeability of TMZ using triolein emulsion injection would make it the most effective chemotherapeutic agent currently available for the treatment of malignant glioma. To the best of our knowledge, no study of the enhancement of the permeability of TMZ in the brain using a triolein emulsion has been reported using HPLC analysis or DESI-MS imaging. Thus, the aim of the present study was to evaluate the difference in the concentration of TMZ in the rat brain with and without the administration of triolein emulsion into the carotid artery using MS techniques.

## Materials and methods

### Drugs, reagents, and surgical material

TMZ, triolein, and trypan blue were purchased from Sigma-Aldrich (St. Louis, MO, USA). Silk and ketamine hydrochloride were purchased from Ailee (Busan, Korea) and Huons (Gyeonggi-do, Korea), respectively, whereas Rompun (xylazine) was purchased from Bayer Korea (Seoul, Korea). Dimethyl sulfoxide (DMSO) was purchased from Daejung (Siheung-si, Korea). Intravenous (IV) catheters and 2-mL syringes were purchased from Dukwoo Medical Co., Ltd. (Gyeonggi-do, Korea) and Korea Vaccine (Seoul, Korea), respectively. The three-way stopcock was purchased from Becton Dickinson (Franklin Lakes, NJ, USA).

### Animal model establishment

The Institutional Animal Review Board of the Biomedical Research Institute approved all experimental protocols (approval no: 2020-009-A1C0). The experiments were performed using 10-week-old male Sprague-Dawley rats (SAMTACO, Osan, Korea) weighing approximately 300 g after 2–3 days of acclimation. All animals were kept in a semi-specific pathogen-free environment maintained at 18–22 °C under a 12-h light/dark cycle and were allowed access to water and food ad libitum. Animals were anesthetized using an intramuscular injection of ketamine hydrochloride (2.5 mg/kg) and xylazine (0.125 mg/kg) and were allowed to breathe ambient air spontaneously during the procedure.

To determine whether triolein enhanced the permeability of TMZ, three groups of rats were used. Group 1 (negative control) was untreated, group 2 and 4 (experimental groups) were treated with triolein and TMZ, and group 3 and 5 (positive control groups) were treated with TMZ alone. The TMZ injection was administered at 20 mg/kg to groups 2 (*n* = 8) and 3 (*n* = 8), and a parallel set of groups 4 (*n* = 8) and 5 (*n* = 8) were administered 10 mg/kg.

### Experimental protocol

**Figure 1.**
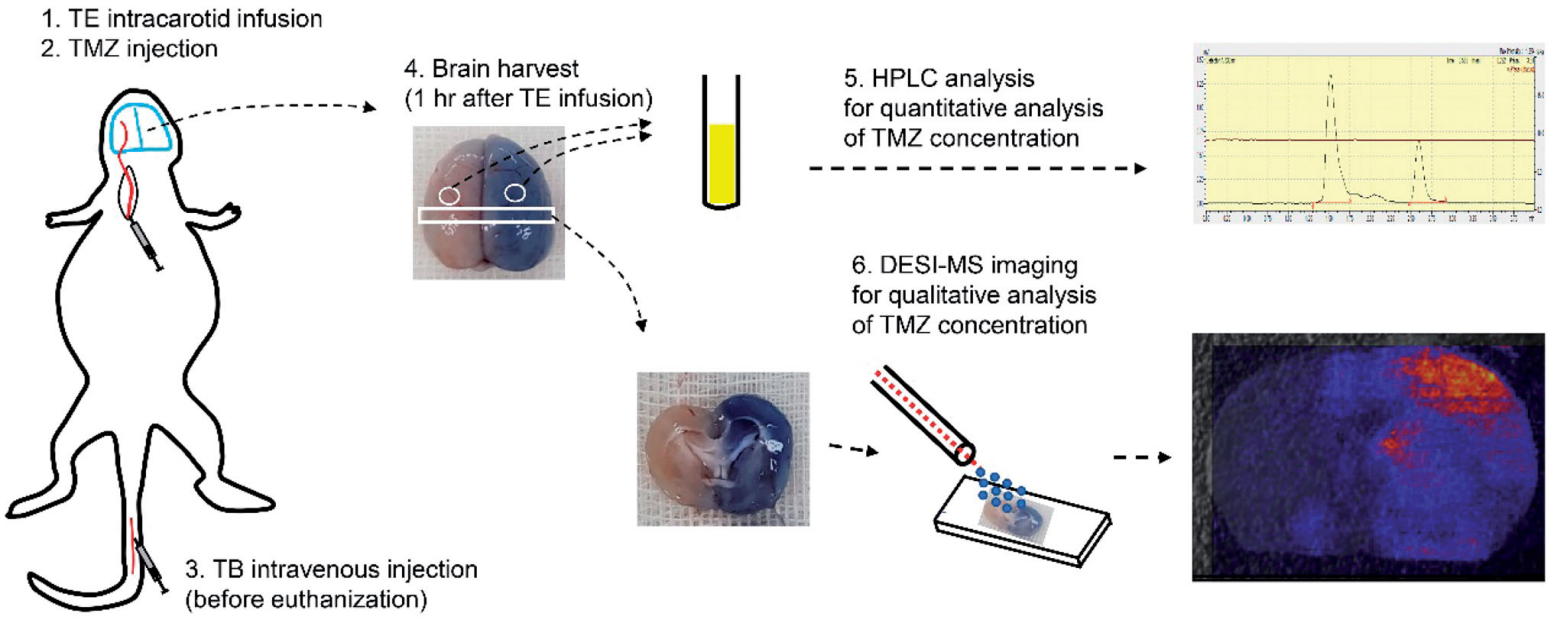
Experimental protocol used in the present study. TE, triolein emulsion; TMZ, temozolomide; TB, trypan blue; HPLC, high-performance liquid chromatography; DESI-MS, desorption electrospray ionization mass spectrometry.

Experimental protocol used in the present study was described as a schema in the Figure 1. First of all, TE was infused into the right common carotid artery. Just after that, TMZ injection was followed. Before euthanization of the rat, trypan blue was injected intravenously at the tail vein. Euthanization of the rats was performed 1 hour after TE infusion using carbon dioxide gas. Brain tissue was obtained and two different studies were performed. One was the experiment for quantitative analysis of TMZ concentration using the HPLC method, and another for qualitative analysis of TMZ concentration using the DESI-MS imaging method.

### Triolein emulsion infusion

The hair on the right cervical area of each rat was removed using an electric razor and a longitudinal incision was made. The right common carotid artery was isolated, and the right external carotid artery was ligated with a silk thread under an operating microscope (Olympus SZX7-STU2, Tokyo, Japan) to ensure that the target drug only passed through the right internal carotid artery. A 24-gauge IV catheter (Angiocath; Becton Dickinson) was inserted into the right common carotid artery. A 1-mL syringe containing 50 μL neutral triglyceride triolein (molecular weight [MW] = 885.5 g/mol) and a 10-mL syringe containing 10 mL normal saline were connected to the three-way stopcock. Triolein emulsion (50 μL/10 mL, 0.05%) was prepared by mixing triolein and normal saline using a vigorous to-and-fro movement of the syringes for 3 min. Triolein emulsion (2 mL) was injected into the right common carotid artery of each rat over a period of 30 s.

### TMZ preparation

TMZ was administered at doses of 20 mg/kg and 10 mg/kg to the respective groups. The TMZ formula was administered intravenously to patients at doses of 50–200 mg/m^2^ as a solution in 3% DMSO in the initial phase I trial. The animals in the present study received 20 and 10 mg/kg TMZ, and a 10 mg/kg rat dose was equivalent to a 150 mg/m^2^ adult human dose (Patel et al., 2003). TMZ was dissolved in DMSO at concentrations of 36 and 18 mg/mL and then further diluted with normal saline. Specifically, 0.167 mL TMZ-DMSO stock was mixed with 1.833 mL normal saline, and a 2-mL solution was prepared immediately before the injection to avoid any possible degradation of TMZ.

### TMZ injection and brain tissue harvesting

The rats were administered 2 mL TMZ via an intracarotid arterial injection (36 and 18 mg/kg) immediately after triolein emulsion infusion. For the gross evaluation of the opening of the BBB, trypan blue (2 mL) was intravenously injected before euthanization. All the rats were euthanized 1 hour after TE infusion using carbon dioxide gas. Trypan blue-stained brain tissue samples were harvested and placed in 10 mL 0.5 M HCl buffer solution for HPLC analysis.

### Quantitative HPLC and statistical analyses

The brain tissue samples were chopped, sonicated for 10 min at 20–25 °C, centrifuged at 3000 ×*g* at 20–25 °C, and then, the supernatants were collected, followed by subsequent centrifugation at 17,000 ×*g* for 30 min and the supernatant was collected for HPLC analysis (Shimadzu, Kyoto, Japan) to determine the concentration of TMZ. The HPLC system was fitted out with an SPD-20A Prominence UV/Vis detector, an LC-20AT liquid chromatograph, a CT-20A Prominence column oven, a DGU-20ASR degassing unit and an SIL-20 Prominence autosampler. A VDSpher^®^ PUR 100 C18-E column (5 µm, 150 × 4.6 mm, VDS Optilab, Berlin, Germany) was used.

The internal standard equation was predetermined and the TMZ concentrations were analyzed in a mobile phase consisting of 8:2 volumes of 0.5% (v/v) acetic acid:methanol at a flow rate of 1.1 mL/min, oven temperature of 40 °C, and wavelength of 330 nm. The internal standard equation for TMZ based on the TMZ concentration and area under the curve (AUC) was calculated using a linear regression method. TMZ quantification was validated using an internal standard equation. The detection range was 0.064–40 µg/mL TMZ and an AUC of 2764.9–1514221.3. The equation of TMZ concentration and AUC was “y = 37815x + 2554.7” and R^2^ = 1. The method was fully validated for precision, accuracy, selectivity, and linearity (Gilant et al., 2012). The AUC values of the TMZ peak at a retention time of 2.480 min were calculated as the concentration of TMZ in the brain. TMZ concentrations were compared between groups, and the TMZ concentration ratios of the right to left hemisphere of each group were calculated. All data analyses were conducted using a two-tailed unpaired t-test using GraphPad Prism 5.0 (GraphPad Software, Inc., La Jolla, CA), and the statistical significance was set at *p* < .05.

### Qualitative analysis using DESI-MS imaging

DESI-MS imaging experiments were performed using a Waters XEVO G2-XS quadrupole time-of-flight (Q-ToF) mass spectrometer (Waters, Milford, MA, USA). The DESI ion source (Waters) was mounted on a mass spectrometer and controlled using Omni Spray software (Prosolia, Indianapolis, IN, USA). The DESI source was initially set up using a rock spray solvent of 0.1% formic acid in acetonitrile:water (95:5, v/v) and 0.2 ng/μL leucine enkephalin (m/z 556.2771 in the ESI + mode) was added as the internal standard and lock mass compound.

The flow rate was 2 μL/min in the positive ion mode. All MS parameters were recorded under the following condition: capillary voltage, 5 keV; sample cone voltage, 40 V; source and desolvation temperatures, 150 °C and 250 °C, respectively; desolvation and cone gas flow rates, 600 L/h and 50 L/h, respectively; gas pressure, 4.5 bar; spray voltage, 4.5 kV; spray, sprayer incidence, and collection angles, 60°, 75°, and 10°, respectively; sprayer-to-inlet and sprayer-to-sample distances, 3 mm and 1 mm, respectively; source temperature, 150 °C; and source offset, 80 V.

Prior to image acquisition, the detected ion intensity of the red Sharpie marker pen (rhodamine) [M + H]^+^ at m/z 443.23 for positive ion mode was verified to conduct the experiment. The emitter capillary protrusion was initially optimized by observing the removal of material from an ink patch on a glass slide. The mass spectra were acquired in the range of m/z 50–700 for all MS analyses. Furthermore, 1000 of the most intense peaks were observed. All m/z values were extracted at a mass window of 0.02 Da. The total scan time was determined based on the pixel size and scan speed.

The square pixel size was 100 μm for the imaging MS, whereas the scan speeds were 100–110 μm/s. The time required for imaging the brain samples was 230–240 min. All imaging data were acquired and analyzed directly using high-definition imaging (HDI) version 1.4 in combination with MassLynx version 4.1 (Waters, Milford, MA, USA). The HDI imaging software enabled the acquisition and processing of data from the DESI-MS imaging experiments.

## Results

**Figure 2.**
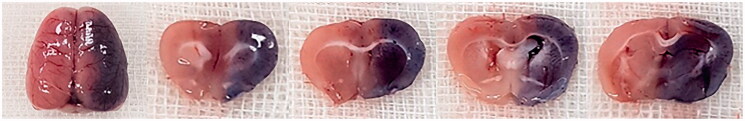
Representative trypan blue-stained brain in group 2 and 4 as a whole and its sections. Ipsilateral hemisphere of shows blue stain due to BBB opening by triolein emulsion. The rats of group 1 (negative control group) or group 3 and 5 (positive control groups) did not reveal staining, however.

The whole brain and tissue sections of the treated hemispheres were stained blue with trypan blue in the experimental groups (group 2 and 4, Figure 2). Some rats showed blue staining of the contralateral hemisphere because of the bilateral supply of the cerebral artery. The rats of the group 1 (negative control group, *n* = 2) or the positive control groups (group 3 and 5) did not reveal staining with trypan blue.

### Quantitative HPLC and statistical analyses

#### TMZ 20 mg/kg treatment

**Figure 3.**
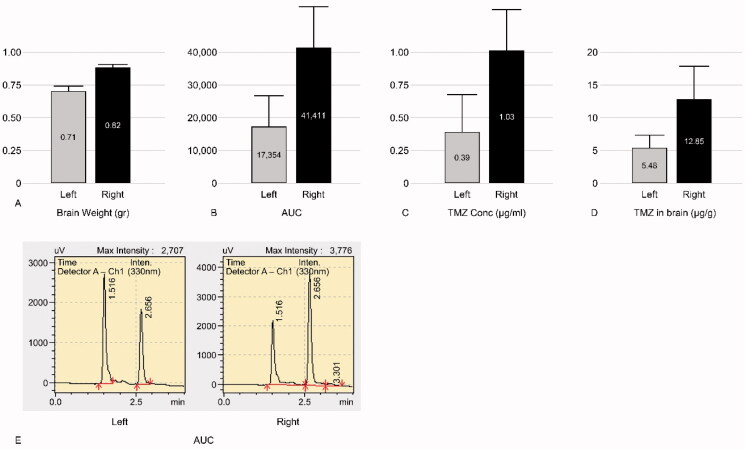
Graphs of experimental group of temozolomide (TMZ) 20 mg/kg injection (group 2). Left, contralateral hemisphere; Right, ipsilateral hemisphere. (A) Mean weight of the tissue obtained from the harvested brain. (B) Mean area under the curve (AUC); AUC of the ipsilateral hemisphere is higher than the contralateral hemisphere. (C) Mean TMZ concentration per 1 mL buffer calculated by the AUC and standard equation (y = 37815x + 2554.7). Mean TMZ concentration per 1 ml buffer of the ipsilateral hemisphere is higher than the contralateral hemisphere. (D) Mean TMZ concentration per 1 gr brain weight calculated from TMZ Conc (C) × 10 ml buffer/Brain weight (A), is higher in the ipsilateral hemisphere than the contralateral hemisphere. (E) A representative mass spectrum of B; the peaks of TMZ and the buffer were observed at 2.65 min and 1.51 min, respectively. The AUC of the ipsilateral hemisphere was wider than the contralateral hemisphere.

**Figure 4.**
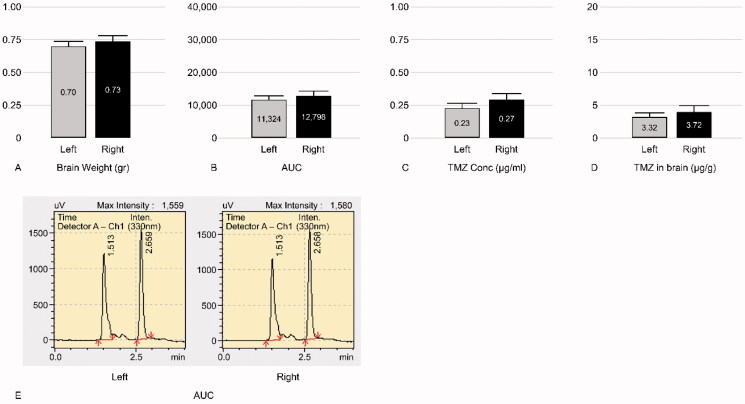
Graphs of control group of TMZ 20 mg/kg injection (group 3). Left, contralateral hemisphere; Right, ipsilateral hemisphere. (A) Mean weight of the tissue obtained from the harvested brain. (B) Mean AUC. (C) Mean TMZ concentration per 1 mL buffer. (D) Mean TMZ concentration per 1 gr brain weight. Mean TMZ concentration ratio ± SD of the ipsilateral hemisphere to the contralateral hemisphere is 1.13 ± 0.14. (E) A representative mass spectrum of B; the peaks of TMZ and the buffer were observed at 2.65 min and 1.51 min, respectively. AUC of the ipsilateral hemisphere is similar to that of the contralateral hemisphere.

**Figure 5.**
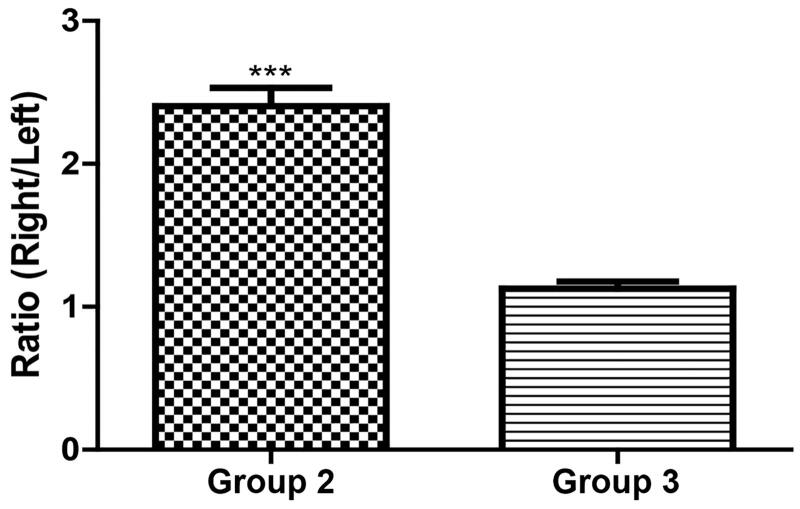
Comparison of TMZ concentration ratio of ipsilateral (right) to contralateral (left) hemispheres of rats in group 2 and 3 treated with 20 mg/kg injection; ***p < .0001, significant difference between groups 2 and 3 using a two-tailed unpaired *t*-test.

TMZ was not detected in the bilateral hemispheres of group 1 rats (negative control group). The mean ± standard deviation (SD) of TMZ concentration was 12.85 ± 5.02 and 5.48 ± 2.56 µg/g in the ipsilateral and contralateral hemispheres of the group 2 rats (experimental group), respectively. The mean ratio ± SD of the ipsilateral hemisphere to the contralateral hemisphere in group 2 was 2.40 ± 0.36 (Figure 3). In group 3 (positive control group), the mean ± SD concentrations of TMZ were 3.72 ± 0.44 and 3.32 ± 0.40 µg/g in the ipsilateral and contralateral hemispheres, respectively. The mean ratio ± SD of the ipsilateral hemisphere to the contralateral hemisphere of group 3 was 1.13 ± 0.14 (Figure 4). Thus, triolein emulsion significantly (two-tailed unpaired *t*-test, *p* < .05) enhanced the delivery of TMZ, which was 2.12 (2.40/1.13) times higher than that of TMZ administered alone (Figure 5).

#### TMZ 10 mg/kg treatment

**Figure 6.**
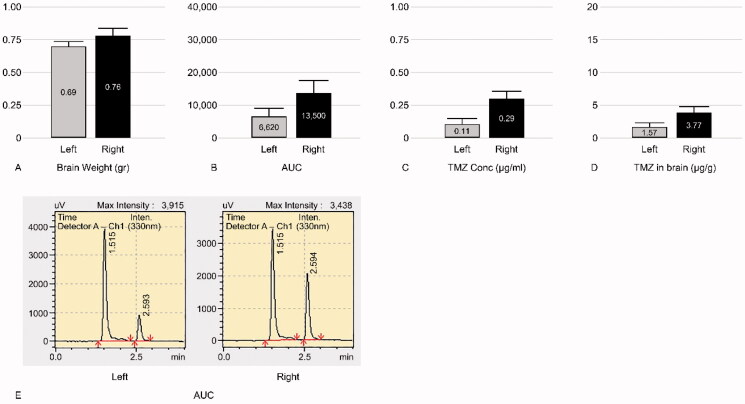
Graphs of experimental group (group 4) of TMZ 10 mg/kg injection. Left, contralateral hemisphere; Right, ipsilateral hemisphere. (A) Mean weight of the tissue obtained from the harvested brain. (B) Mean AUC. (C) Mean TMZ concentration per 1 ml buffer. (D) Mean TMZ concentration per 1 gr brain weight. Mean TMZ concentration ratio ± SD of the ipsilateral hemisphere to the contralateral hemisphere is 2.54 ± 0.47. (E) A representative mass spectrum of B; the peaks of TMZ and the buffer were observed at 2.65 min and 1.51 min, respectively. AUC of the ipsilateral hemisphere is wider than the contralateral hemisphere.

**Figure 7.**
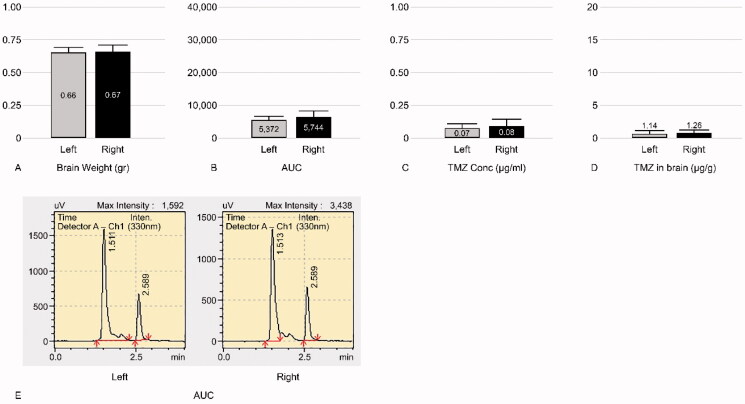
Graphs of control group (group 5) of TMZ 10 mg/kg injection. Left, contralateral hemisphere; Right, ipsilateral hemisphere. (A) Mean weight of the tissue obtained from the harvested brain. (B) Mean AUC. (C) Mean TMZ concentration per 1 mL buffer. (D) Mean TMZ concentration per 1 gr brain weight. Mean TMZ concentration ratio ± SD of the ipsilateral hemisphere to the contralateral hemisphere was 1.11 ± 0.09. (E) A representative mass spectrum of B; the peaks of TMZ and the buffer were observed at 2.65 min and 1.51 min, respectively. AUC of the ipsilateral hemisphere is similar to that of the contralateral hemisphere.

**Figure 8.**
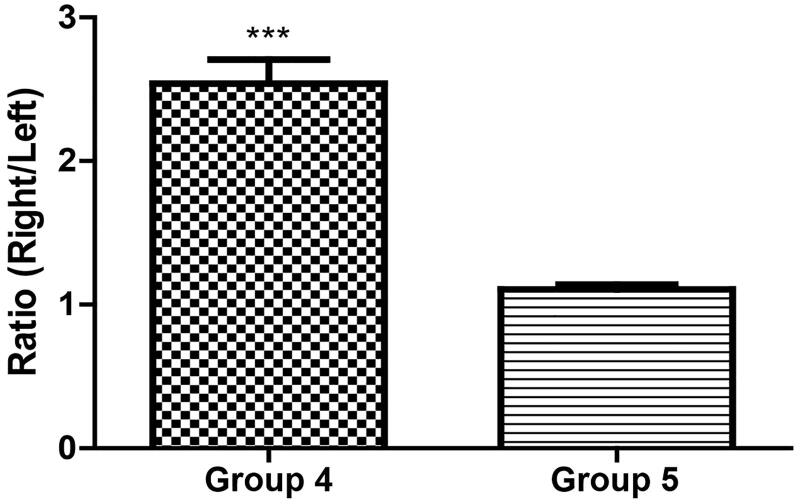
Comparison of TMZ concentration ratio of ipsilateral (right) to contralateral (left) hemispheres of rats in group 4 and 5 injected with 10 mg/kg; ***p < .0001, significant difference between groups 4 and 5 using a two-tailed unpaired *t*-test.

The mean ± SD of TMZ concentration was 3.77 ± 1.32 µg/g and 1.57 ± 0.76 µg/g in the treated and contralateral hemispheres of the group 4 rats (experimental group), respectively. Thus, the mean ratio ± SD of the ipsilateral hemisphere to the contralateral hemisphere in group 4 was 2.54 ± 0.47 (Figure 6). In group 5 (positive control group), the mean ± SD of TMZ concentration was 1.26 ± 0.26 µg/g and 1.14 ± 0.21 µg/g in the ipsilateral and contralateral hemispheres, respectively. The concentration ratio ± SD of the ipsilateral hemisphere to the contralateral hemisphere in group 5 was 1.11 ± 0.09 (Figure 7). Thus, in the presence of triolein emulsion, the delivery of TMZ was significantly (two-tailed unpaired *t*-test, *p* < .05) higher (2.28, 2.54/1.11 times) than that observed without the emulsion (Figure 8).

### Qualitative analysis (DESI-MS imaging for TMZ concentration)

**Figure 9.**
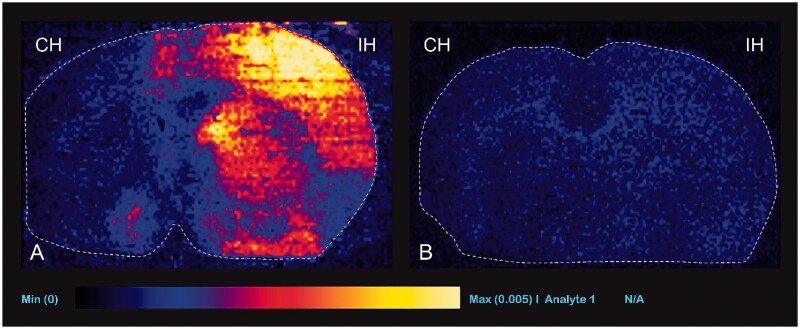
Representative desorption electrospray ionization mass spectrometry images with TMZ 20 mg/kg injection. Ipsilateral (IH, right) hemisphere shows higher signal intensity than the contralateral (CH, left) hemisphere in experimental group (group 2, A) and positive control group (group 3, B).

**Figure 10.**
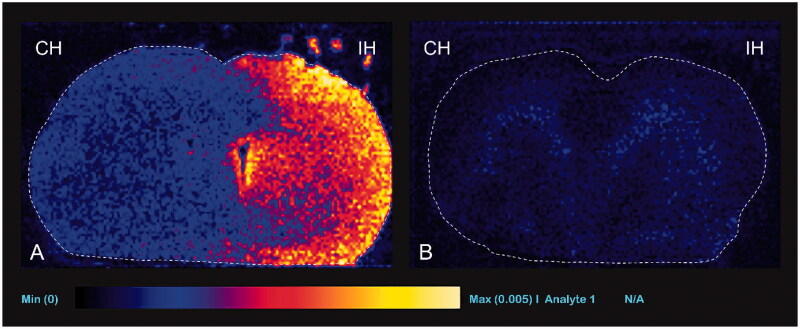
Representative desorption electrospray ionization mass spectrometry images with temozolomide 10 mg/kg injection. Ipsilateral (IH, right) hemisphere shows higher signal intensity than the contralateral (CH, left) hemisphere (group 4, A) and positive control group (group 5, B).

TMZ was identified at m/z 217 because of the sodium adduct ions [M + Na]^+^ 194 → 217. The signal intensity of TMZ was not detected using DESI-MS imaging in group 1 (negative control group). In group 2 and 4 (experimental groups), the signal intensity of TMZ was remarkably high in the ipsilateral hemisphere (Figures 9(A) and 10(A)). In group 3 and 5 (positive control groups), the ipsilateral hemisphere showed minimal or no TMZ signal (Figures 9(B) and 10(B)). The signal intensities of the rats treated with TMZ 20 mg/kg (group 2) tended to be higher than those of the TMZ 10 mg/kg treated rats (group 4).

## Discussion

In the present study, we focused on evaluating the enhancement of TMZ delivery in the rat brain by co-administration of triolein emulsion infusion through the carotid artery. The concentration of TMZ was approximately two times higher in the rat brain in the presence of the triolein emulsion than it was without the emulsion. The ratios of the TMZ concentration in the ipsilateral hemisphere to that in the contralateral hemisphere were 2.13 and 2.18 times higher following the injections of TMZ 20 mg/kg and 10 mg/kg, respectively, than they were before treatment and were significantly different from those of the control group. These results indicate that the vascular permeability of the brain was significantly increased after triolein emulsion was injected into the cerebral artery.

Triolein, a major constituent of the bone marrow and the main cause of clinical fat embolism, induces vasogenic edema immediately after it is injected into the carotid artery (Kim et al., 2001). Cerebral infarction may occur when triolein is administered as a bolus injection, but not when it is injected as an emulsion (Kim et al., 2004). Thus, the development of cerebral fat embolisms depends on the morphology of the embolized fat and the size of the fat particles. The increased vasogenic edema induced by triolein emulsion is reversible and does not cause any significant histological changes, hemodynamic occlusive appearance, or metabolic differences in the brain (Kim et al., 2004, 2006; Baik et al., 2008).

The reversible and temporal increase in vasogenic edema is because of the increased vascular permeability of the cerebral artery and can be useful in the adjuvant treatment of intractable brain diseases, such as malignant tumors, encephalopathy, epilepsy, and neurodegenerative disorders. The molecular mechanism mediating the increase in vascular permeability induced by triolein emulsion is unknown, and the morphological pathway mainly involves tight junctions and minimal transcytosis in the cerebral endothelium (Sol et al., 2017). Recently, a study using triolein emulsion infusion into the hepatic arteries was reported to increase the doxorubicin concentration approximately twofold and suggests that a triolein infusion might be a useful adjuvant treatment of liver cancer (Kim et al., 2021).

Currently, despite aggressive combination treatments, the prognosis of malignant glioma remains poor because of the unique structure of the brain. In particularly, the BBB, which consists of endothelial cells with tight junctions lining the microvasculature of the brain, impedes the penetration of drugs. TMZ, which is the only chemotherapeutic agent that improves the survival rate of patients with malignant gliomas, is lipophilic and penetrates the BBB (Brada et al., 1999). The penetration of TMZ administered orally or intravenously into the brain has been shown to be variable (20%–39%) based on the brain/plasma AUC ratio (Reyderman et al., 2004; Ostermann et al., 2004).

TMZ exerts chemotherapeutic activity following its conversion to linear triazine MTIC, which is known to have an important chemotherapeutic effect mediated by a potent alkylating activity. TMZ inserts a methyl group into the purines and pyrimidines of the DNA, resulting in cell death (Zhang et al., 2012). The 2-year survival rate of patients with malignant gliomas was slightly higher at 24% with combined radiation and TMZ therapy than it was at 10% with radiation treatment alone (Stupp et al., 2005).

Several new techniques have been investigated for promoting the delivery of TMZ through the BBB using MS for quantification in animal models (Zhang et al., 2012; Stupp et al., 2005). Focused ultrasound (FUS) has recently been used to overcome the challenges of penetrating the BBB and enhances the delivery of anticancer agents (Zhang et al., 2012). After oral administration of TMZ (50 mg/kg), FUS enhanced the penetration in mouse brains by 2.7 times the levels observed in the group administered TMZ alone (Liu et al., 2014). Regadenoson, which is used as a cardiac stress agent in patients who are unable to walk on a treadmill test, has also been used to transiently disrupt the BBB, which subsequently increases TMZ levels in the rat brain (Stupp et al., 2005). The brain TMZ concentration after oral administration of 50 mg/kg was 1.59 times higher in the presence of regadenoson than it was with TMZ alone (Jackson et al., 2016). These results are quite similar to those of the present study, although the administration route and amount of TMZ were different.

HPLC and LC-MS/MS are the most common and validated techniques for the analysis of TMZ concentrations in animal models and patients (Patel et al., 2003; Brada et al., 1999; Ostermann et al., 2004; Kim et al., 2001; Diez et al., 2010). Furthermore, studies using these techniques have shown delayed TMZ peak concentration at 2.5 h and a mean half-life of 1.5 h after infusion through the cerebrospinal fluid (CSF). The delivery of TMZ from the plasma to the CSF was substantial (CSF:plasma AUC ratio, 0.33) (Patel et al., 2003). MTIC, the active form of TMZ, is produced by chemical degradation at physiological pH. MTIC forms methyl adducts at the N^7^-position of guanine, N^3^-position of adenine, and O^6^-position of guanine. Furthermore, O^6^-methylguanine appears to be a critical cytotoxic molecule (Gibson et al., 1984). MTIC is then degraded to the inactive derivatives 5-aminoimidazole-4-carboxamide and methyldiazonium cation (Marchesi et al., 2007).

However, prolonged treatment results in resistance and low efficacy of subsequent therapy because of DNA damage repair enzymes, which leads to the recurrence of malignancy in 60%–75% of patients (Chamberlain, 2010; Beier et al., 2011). For successful treatment, high doses of TMZ may be needed, which results in increased toxicity and several complications. To overcome the shortcomings of TMZ therapy, a delivery carrier is required to increase its effects at the target site. Several carriers of TMZ have been reported, but they lack tumor-specific delivery (Tentori & Graziani, 2009). An efficient drug delivery system for glioma therapy should target the tumor and be able to cross the BBB.

The qualitative analysis of TMZ concentration in the present study showed a remarkably higher signal intensity in the treated hemispheres than in the contralateral hemispheres and control groups. In addition, the signal intensity tended to be stronger after higher amounts of TMZ were injected. The signal intensity observed with DESI-MS imaging correlated with the TMZ concentrations determined using HPLC. Thus, DESI-MS imaging appeared to be suitable for determining TMZ concentration levels in tissues and organs. In the control groups, the signal intensity was not changed or was slightly increased in the treated hemispheres, depending on the acquisition scale. The mild signal intensity detected using DESI-MS imaging in the control group in the present study indicated that TMZ was delivered at normal levels to the brain parenchyma through the BBB. The concentration in the brain parenchyma was nearly 30% of the plasma concentration (Agarwala & Kirkwood, 2000). Presumably, this is the first study to report the use of HPLC analysis and DESI-MS imaging of TMZ. DESI-MS imaging is an emerging and powerful technique that has been recently developed for the imaging of lipids or metabolites in tissues (Takats et al., 2004; Wiseman et al., 2005). Clinically, this technique has been used to identify malignant tissues in brain, breast, or prostate cancers (Eberlin et al., 2011; Santagata et al., 2014; Kerian et al., 2015; Calligaris et al., 2014). The outstanding advantage of the DESI-MS technique is the time efficiency gained because no complex sample preparation or separation techniques, such as extraction and chromatography, is required. In addition, this technique has high discriminating power, sensitivity, specificity, and the throughput and ability to analyze specimens in a wide mass spectrum from simple amino acids to drug molecules, alkaloids, terpenoids, and steroids to peptides and proteins (Cooks et al., 2006; Lostun et al., 2015). The technique is also extremely efficient for surface analysis, mapping, and forensic applications, especially because of it has minimal destructiveness and enables possible in-situ measurements (Takats et al., 2004; Cooks et al., 2006).

The present technique could be applicable in many clinical fields, such as malignant brain tumors of primary or metastatic cancers, retinal or testicular malignancies under conditions of having blood-tissue barriers, refractory hepatic or pancreatic malignancies, of the hypovascular conditions showing difficulty in chemotherapy. Enhancement of drug delivery by triolein emulsion is independent of the organs that the technique was used (Kim et al., 2004, 2009, 2016, 2021; Lee et al., 2009).

In conclusion, the results of the present study demonstrate that triolein emulsion enhances the penetrability of the BBB, thereby providing a potential new strategy to enhance the therapeutic effect of TMZ. Furthermore, HPLC and DESI-MS imaging are suitable for the quantitative and qualitative analyses, respectively, of TMZ concentrations in brain tissue.
